# Source of ICU admission: does it really matter?

**DOI:** 10.1186/cc14642

**Published:** 2015-03-16

**Authors:** A Datta, A Kar, A Ahmed

**Affiliations:** 1 Medica Superspecialty Hospital, Kolkata, India

## Introduction

Source of admission to the ICU is of importance. We tried to identify the different sources of ICU admission to a level 3 ICU of a tertiary care hospital in Kolkata and analyze whether the overall patient outcome is affected by the different sources of admission.

## Methods

Our prospective study included 2,056 patients admitted to a level 3 care ICU over a period of 2 years. Numbers of readmissions were not considered. ICU outcome was analyzed using the APACHE IV model and source of admission to the ICU was documented as either from emergency (ER), from the floor or from other hospital. Analysis was carried out between different groups based on admission using unpaired Student *t *test and *P *< 0.05 was considered significant. Number of ventilations and the mortality rate in each group were also documented.

## Results

Of 2,056 admissions, 1,223 cases (59.48%) were from ER, 809 cases (39.35%) were from floor and 24 cases (1.16%) were from other hospitals. In the ER group, mean APACHE IV was 55.03 ± 31.49 SD (median 50), PMR 16.26, observed deaths 198, ALOS 4.78 days ± 4.83 SD (median 3), SMR 0.995 (CI = 0.86 to 1.14), mean age 60.52 years ± 17.63 SD (median 63), 323 ventilations. In the floor group, mean APACHE IV was 65.17 ± 34.40 SD (median 60), PMR 27.03, observed deaths 234, ALOS 5.23 days ± 5.22 SD (median 3), SMR 1.07 (CI = 0.94 to 1.21), mean age 61.38 years ± 15.72 SD (median 64), 302 ventilations. In the other hospital group, mean APACHE IV was 55.29 ± 29.82 SD (median 50), PMR 18.0, observed deaths 2, ALOS 6 days ± 5.85 SD (median 3), SMR 0.46 (CI: 0.23 to 0.88), mean age 56.08 years ± 17.79 SD (median 56.5), six ventilations. During analysis, the other hospital group was omitted because of inadequate sample size. There was statistically significant differences in APACHE IV (floor >ER, *P *< 0.0001), PMR (floor >ER, *P *< 0.0001), ALOS (floor >ER, *P *= 0.04) noted between the floor and ER groups. Number of ventilations (37.33% vs. 26.4%), SMR (1.07 vs. 0.995), and mortality rate (28.92% vs. 16.19%) were also significantly higher for patients admitted from the floor. No significant statistical difference was observed in age between two groups (*P *= 0.26).

## Conclusion

The severity of illness index in patients admitted to the ICU from floors is significantly higher than emergency admissions. Overall outcome for patients transferred to the ICU from the floor is worse based on mortality rate, SMR, and ALOS when compared with the emergency group.

